# Distinctive features of single nucleotide alterations in induced pluripotent stem cells with different types of DNA repair deficiency disorders

**DOI:** 10.1038/srep26342

**Published:** 2016-05-20

**Authors:** Kohji Okamura, Hironari Sakaguchi, Rie Sakamoto-Abutani, Mahito Nakanishi, Ken Nishimura, Mayu Yamazaki-Inoue, Manami Ohtaka, Vaiyapuri Subbarayan Periasamy, Ali Abdullah Alshatwi, Akon Higuchi, Kazunori Hanaoka, Kazuhiko Nakabayashi, Shuji Takada, Kenichiro Hata, Masashi Toyoda, Akihiro Umezawa

**Affiliations:** 1 Department of Systems BioMedicine, National Research Institute for Child Health and Development, Tokyo, 157-8535, Japan; 2 Department of Reproduction, National Research Institute for Child Health and Development, Tokyo, 157-8535, Japan; 3 Biotechnology Research Institute for Drug Discovery, National Institute of Advanced Industrial Science and Technology (AIST), Ibaraki, 305-8568, Japan; 4 Laboratory of Gene Regulation, Faculty of Medicine, University of Tsukuba, Ibaraki, 305-8575, Japan; 5 Nanobiotechnology and Molecular Biology Research Lab, Department of Food Science and Nutrition, College of Food Science and Agriculture, King Saud University, P.O. Box 2460, Riyadh, 11451, Saudi Arabia; 6 Department of Food Sciences and Nutrition, College of Food and Agricultural Sciences, King Saud University, P.O. Box 2460, Riyadh 11451, Saudi Arabia; 7 Department of Chemical and Materials Engineering, National Central University, No. 300, Jhongda RD., Jhongli, Taoyuan 32001, Taiwan; 8 College of Science, King Saud University, P.O. Box 2455, Riyadh 11451, Saudi Arabia; 9 Department of BioSciences, Kitasato University School of Science, Kanagawa, 252-0373, Japan; 10 Department of Maternal-Fetal Biology, National Research Institute for Child Health and Development, Tokyo, 157-8535, Japan; 11 Research team for Geriatric Medicine (Vascular Medicine), Tokyo Metropolitan Institute of Gerontology, Tokyo, 173-0015, Japan

## Abstract

Disease-specific induced pluripotent stem cells (iPSCs) have been used as a model to analyze pathogenesis of disease. In this study, we generated iPSCs derived from a fibroblastic cell line of xeroderma pigmentosum (XP) group A (XPA-iPSCs), a rare autosomal recessive hereditary disease in which patients develop skin cancer in the areas of skin exposed to sunlight. XPA-iPSCs exhibited hypersensitivity to ultraviolet exposure and accumulation of single-nucleotide substitutions when compared with ataxia telangiectasia-derived iPSCs that were established in a previous study. However, XPA-iPSCs did not show any chromosomal instability *in vitro*, i.e. intact chromosomes were maintained. The results were mutually compensating for examining two major sources of mutations, nucleotide excision repair deficiency and double-strand break repair deficiency. Like XP patients, XPA-iPSCs accumulated single-nucleotide substitutions that are associated with malignant melanoma, a manifestation of XP. These results indicate that XPA-iPSCs may serve a monitoring tool (analogous to the Ames test but using mammalian cells) to measure single-nucleotide alterations, and may be a good model to clarify pathogenesis of XP. In addition, XPA-iPSCs may allow us to facilitate development of drugs that delay genetic alteration and decrease hypersensitivity to ultraviolet for therapeutic applications.

Human induced pluripotent stem cells (iPSCs) impact numerous medical fields including clinical therapy development, drug discovery, research on inherited diseases and studies on reprogramming of differentiated cells^1^,^2^,^3^,^4^. Human iPSCs may also prove valuable for toxicology testing. For example, iPSC-derived hepatocytes have been shown to serve as an *in vitro* tool for understanding drug metabolism and toxicology^5^,^6^. Additionally, iPSCs may be employed to detect the mutagenic potential of chemicals.

Genotoxicity assays assess DNA damage such as single- or double-strand breaks, crosslinking, and point mutations. The Ames test, a bacteria-based assay, is used to detect the mutagenic activity of chemical compounds^7^. Human cells can also be investigated for single nucleotide alterations and indels by genome-wide sequencing analysis. However, each single nucleotide alteration (SNA)/mutation usually occurs in the independent manner or mosaic manner^8^. Unless the mutated cell attains proliferative predominance, the single nucleotide alteration/mutation cannot reach a detectable level. After exposure to mutagens, the cells are highly heterogeneous in terms of mutations. Since iPSCs can be easily subcloned and assessed as a homogeneous population, they may be the key to development of next-generation genotoxicity tests.

iPSCs from healthy donors and patients with DNA repair deficiency disorders can be analyzed for genotoxicity^9^. DNA repair is categorized into two categories, nucleotide excision repair and double-strand break repair, and the molecular mechanisms have been elucidated in detail. Xeroderma pigmentosum (XP) and ataxia telangiectasia (AT) are diseases that exhibit nucleotide excision repair deficiency and double-strand break repair deficiency, respectively. AT-derived iPSCs have been generated and analyzed for chromosomal stability and nucleotide substation rate^9^. In this study, we generated iPSCs from patients with XP group A (XPA-iPSCs) and investigated numbers and types of detected SNAs in mutation-prone iPSCs, which may lead to a novel *in vitro* genotoxicity test using human iPSCs.

## Results

### Generation and characterization of XPA-iPSCs

We generated iPSCs from human cells with a mutation in the *XPA* gene (XP3OS and XPEMB-1 cells) by Sendai virus infection-mediated expression of OCT4/3, SOX2, KLF4, and c-MYC (Supplemental Figure S1A)^10^. When the reprogramming factors OCT4/3, SOX2, KLF4 and c-MYC were introduced into 2.0 × 10^5^ XP3OS and XPEMB-1 cells, iPSCs from each XP cell were successfully generated and designated as XPAiPS-O1 and XPAiPS-E3, respectively. The efficiency of the iPSC colony generation was low compared to that of human intact cells from various adult tissues^11^,^12^,^13^. Morphological characteristics of XP cell-derived iPSCs (XPA-iPSCs), *i.e.* flat and aggregated colonies, were similar to those of other intact iPSCs and ESCs (Supplemental Figure S1B). RT-PCR analysis revealed elimination of the Sendai virus. Southern blot analysis with cDNA probes for each four transgene (*OCT4/3, SOX2, KLF-4,* and *c-MYC*) confirmed that XPAiPS-O1 cells did not have chromosomal integration of the exogenously infected genes (Supplemental Figure S1C). Bands derived from paralogous genes and pseudogenes were also detected under our experimental conditions^14^. Short tandem repeat (STR) analysis showed clonality between the respective iPSC lines and their parental cells (Supplemental Figure S1D). XPAiPS-O1 cells showed an intact karyotype (Supplemental Figure S2). Immunocytochemical analyses demonstrated expression of the pluripotent cell-specific markers, *i.e.* SSEA-4, TRA-1–60, SOX2, NANOG, and OCT4/3, which was consistent with the profile observed in hESCs (Supplemental Figure S3).

To address whether the XPA-iPSCs have the competence to differentiate into specific tissues, teratoma formation was induced by implantation of XPA-iPSCs in the subcutaneous tissue of immunodeficient NOD/SCID mice. XPA-iPSCs produced teratomas within 6–10 weeks after implantation. Histological analysis of paraffin-embedded sections demonstrated that the three primary germ layers were generated as shown by the presence of ectodermal, mesodermal, and endodermal tissues in the teratoma (Supplemental Figure S4), implying the parental cells and XPAiPS-O1 and XPAiPS-E3 cells had potential for multi-lineage differentiation *in vivo*.

### Detection of single-nucleotide and indel mutations

Genomic DNA was extracted from XP3OS, XPAiPS-O1, XPEMB-1, and XPAiPS-E3 samples and fragmented to prepare libraries. The bait sequences employed to enrich exon and non-coding sequences covered approximately 80 Mb and had ability to capture neighbouring regions (see Materials and Methods). The size of the captured regions was much larger than in common exome analyses, which is one of the novel features of the present study. Captured DNA samples were sequenced using Illumina HiSeq 1000. After trimming of adaptor sequences and low-quality bases at both ends, the libraries of XP3OS, XPAiPS-O1, XPEMB-1, and XPAiPS-E3 yielded 16.7 Gb (84.2 million read pairs), 16.9 Gb (85.1 million read pairs), 16.9 Gb (85.4 million read pairs), and 16.6 Gb (83.9 million read pairs), respectively. Paired reads were mapped to the human reference genome to detect mutations as variants. Ratios of PCR duplicates were 0.255, 0.254, 0.281, and 0.193, respectively. After removing the duplicated reads, the mean mapped depths of coverage were 84.2, 85.5, 82.9, and 90.5, respectively.

**Figure 1:**
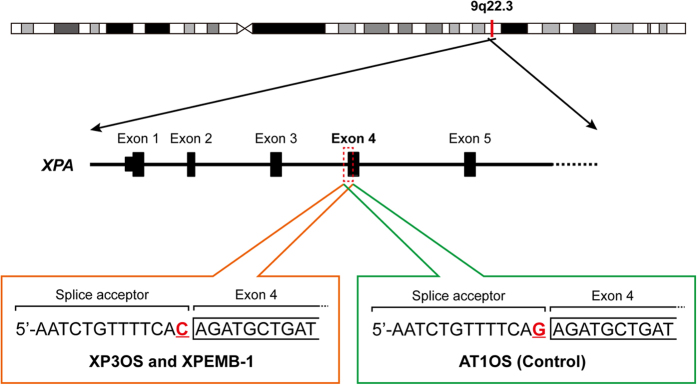
Homozygous mutation at a splice acceptor site of the *XPA* gene in XP3OS and XPEMB-1 parental cells. The *XPA* gene is located on the reverse strand of 9q22.3. It has already been reported that the splice acceptor adjacent to the exon 4 is mutated in both XP3OS and XPEMB-1. The present whole-exome analysis confirmed the mutation from G to C in the both cell lines and their corresponding iPSCs, but not in AT1OS. As for XP3OS and AT1OS, all of the 8 and 28 reads mapped to the region showed C and G at the position, respectively, suggesting that the mutation is homozygous.

To confirm the diagnosis of xeroderma pigmentosum (XP), we first searched all the called single-nucleotide mutations and indel (insertions and/or deletions) variants obtained by comparison with the GRCh37 reference genome for mutations in XP-related genes (Fig. 1 and Supplemental Figure S5)^15^. All four samples, i.e. XP3OS, XPEMB-1, XPAiPS-O1, and XPAiPS-E3 cells, were shown to bear a homozygous mutation at a splice acceptor site of the *XPA* gene (chr9:100,449,544 or NM_000380.3:c.507-1G>C). The mutation was reported to create two abnormally spliced mRNA forms and impair the function of this gene^16^. Because no other mutations were detected in the XP-related genes, this mutation can be assumed to be the cause of the phenotype. The detection of this mutation also confirmed reliability of the present genomic analysis.

**Figure 2:**
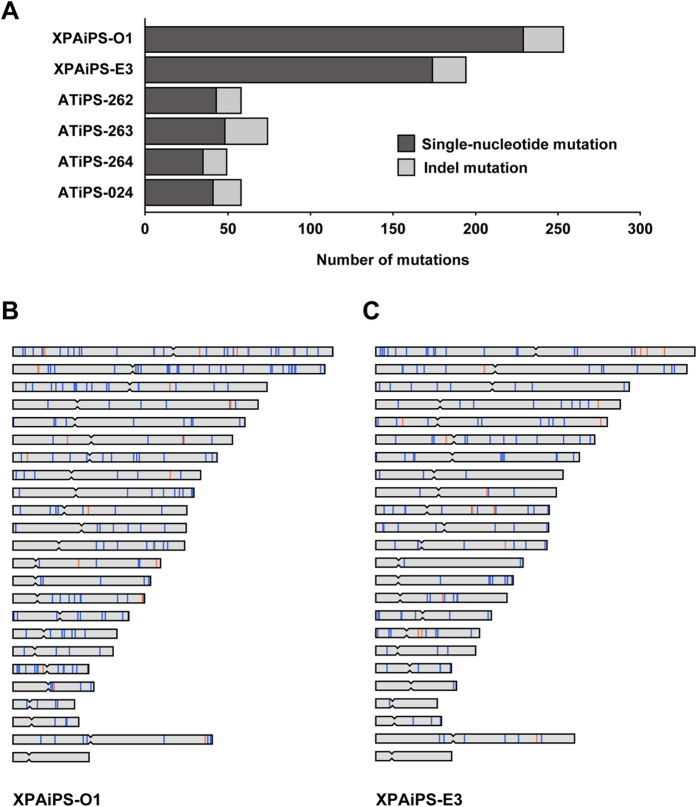
Single-nucleotide and indel mutations identified in XPA-iPSCs. (**A**) Numbers of mutations identified in the present study. The left and right parts of a horizontal bar indicate the numbers of single-nucleotide and indel mutations, respectively. The numbers are also tabulated in Table 1. IDs of iPSC lines are shown on the left. While the two XPA-deficient iPSC lines are derived from two distinct patient samples, the four ATM-deficient iPSC lines are derived from a single patient sample. B and C. Genome-wide distribution of mutations identified in XPAiPS-O1 (**B**) and XPAiPS-E3 (**C**) Positions of single-nucleotide and indel mutations are schematically shown in blue and orange, respectively. Positional occurrence of mutations seems to be random. We did not find mutation hot spots or shared sites between the two iPSC lines.

We, next, compared variants between the parental cells (precursors) and corresponding iPSCs in order to identify mutations that had occurred during the production of iPSCs and subsequent cell cultivation (Fig. 2A). It is noteworthy that method-specific errors were fortunately offset or ignored in this analysis. These mutations are expected to have little possibility to be homozygous. Accordingly, 229 single-nucleotide and 24 indel mutations were detected as *de novo* heterozygous mutations in comparison of the XP3OS and XPAiPS-O1 (Supplemental Table S1). For XPEMB-1, there were 174 single-nucleotide mutations and 20 indels (Supplemental Table S2). The predisposition or hot spots of the mutations in chromosome were detected in neither XPAiPS-O1 nor XPAiPS-E3 cells (Fig. 2B,C).

**Figure 3:**
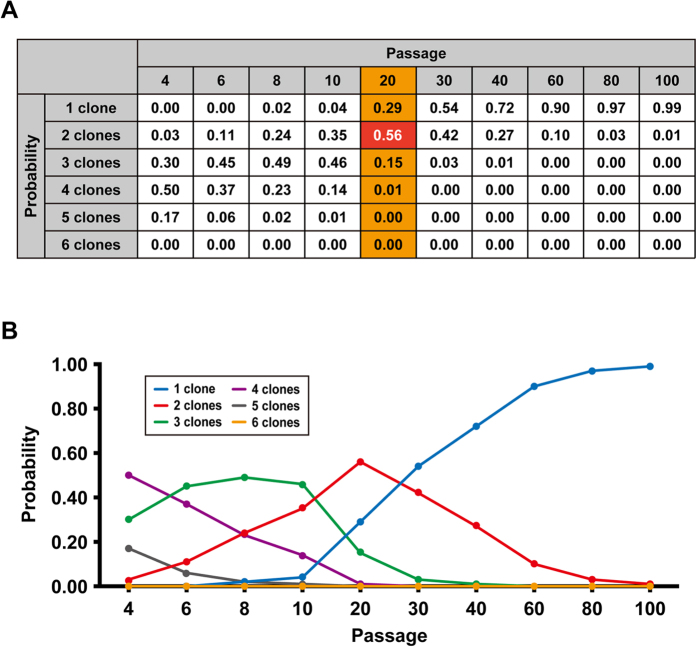
Simulation of clone numbers during passages of iPSCs. To predict the clone numbers of iPSCs, computer simulations were performed according to the experimental conditions described in this study. The conditions of simulation are as follows: 1. The starting condition provides 140 colonies with independent 20 iPSC clones (Passage 0); 2. Twenty colonies are picked and each colony is divided into 7 colonies; 3. Random picking of colonies is repeated at the indicated passage number; 4. Clone number at each passage is counted. Probability of clone numbers at each passage was calculated after the simulation trial was repeated 10,000 times. The probabilities are shown in panels A and B as a table and line plot, respectively.

To verify detection of mutations in iPSCs by whole exome analysis, cell clonality within the same variant was investigated. To this end, a computer simulation was performed with the experimental conditions employed in this study (Fig. 3). The results clearly reveal that 85% of the iPSCs were from one or two clones after 20 passages, suggesting that mutations which had occurred at Passage 0 reached detectable level by the exome analysis.

### Structural alteration in XPA-iPSCs

We also performed a structural alteration analysis using a SNP genotyping microarray for the two pairs consisting of the precursor and corresponding iPSCs. This analysis is also critical for removal of counterfeit calls of single-nucleotide variants that are caused by structural alteration. Whereas, in our previous study, 12 structural mutations including deletion, copy-number gain, and copy neutral LOH were identified in total of four *ATM*-deficient iPSC samples^9^, no structural mutations were detected in the present study using *XPA*-deficient iPSCs. Intriguingly, a significant difference was observed in the numbers of mutation types, *i.e.* either single-nucleotide or indel mutations. Because the numbers of single-nucleotide mutations in AT1OS-derived iPSCs were 43, 48, 35, and 41, XPA-iPSCs have a 4.8-fold increased number of single-nucleotide mutations (Table 1). In contrast, for mutations caused by insertions or deletions, the indel mutations accounted for 30.1% and 9.8% of all detected mutations in the *ATM*-deficient and the *XPA*-deficient iPSCs, respectively. It is highly likely that the *XPA*-deficient iPSCs have a high mutation number of single nucleotide but not of other mutations (Fisher’s exact test, *p* = 5.2 × 10^−11^), indicating the usefulness of the iPSCs for medical research of the disease.

**Figure 4:**
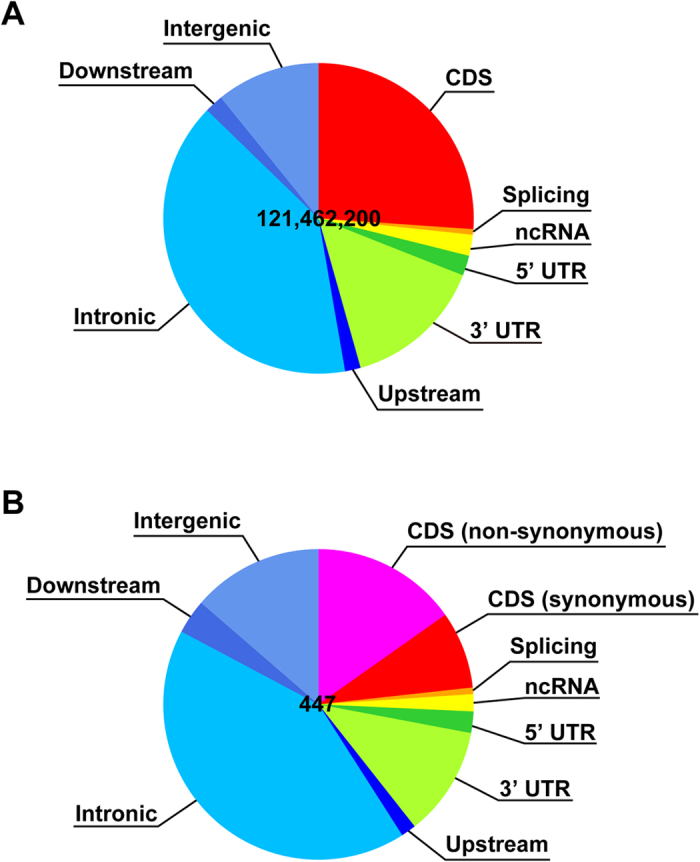
Gene-based annotation. (**A**) While 80-Mb bait sequences in total were used to enrich exonic fragments, variant call was carried out targeting 121.46-Mb regions according to the manufacturer’s instruction. Basically, both of the 100-bp sequences adjacent to each bait were added for the analysis. Annotated results of all of the 121.46 million sites are illustrated as a pie chart. (**B**) Annotated results of the 447 mutated sites identified in XP3AiPS-O1 and XPAiPS-E3 are also illustrated in the same way. The mutation number in protein-coding sequences was slightly lower than that of non-coding sequences, but they seem to be comparable. Mutations might have occurred randomly, and it is unlikely that selection affected the cell population much. It is also supported by the fact that the number of non-synonymous mutations was twice as large as that of synonymous mutations. Using bait covering UTRs and ncRNAs is one of the features of the present exome sequencing. The annotations were performed using ANNOVAR^33^. The details of the gene-based annotation is described on the following website: http://www.openbioinformatics.org/annovar/annovar_gene.html.

Gene-based annotation was performed for the mutations in the 121-Mb target regions (Fig. 4). From the annotated result of the 447 mutated sites identified in XPAiPS-O1 and XPAiPS-E3 cells, the mutation numbers in protein-coding sequences and non-coding sequences were comparable, implying that mutations occur randomly, and that selection seldom affects the cell population much.

**Figure 5:**
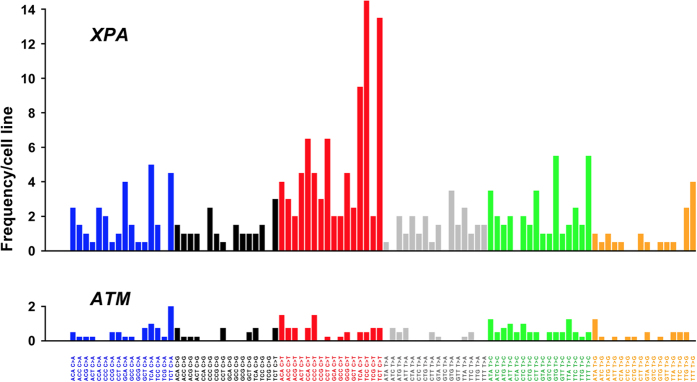
Characteristic patterns observed in combinations of mutation types based on a sequence context. When the trinucleotide sequences in which the mutated base is situated in the center are considered, there are 96 possible mutation types^17^. A combination of these mutation types is known to show a signature of the major mutation process in the cell environment. In the XPA-deficient cell lines, C-to-T or G-to-A mutations were seen at a higher rate compared to other types of mutations. Most of them occurred in a dipyrimidine context. In the ATM-deficient cell lines, the mutation number was low and apparent differences were not observed among mutation types. Vertical axes indicate number of single-nucleotide mutations per cell line.

It has been reported that each tumor shows a characteristic mutation pattern based on a nucleotide context. When the trinucleotide sequences in which the mutated base is situated in the centre are considered, the mutation types are grouped into the specific tumor-categories^17^. From the iPSC mutation patterns in this study, the mutation signature of XPA-iPSCs is suggested to resemble to Signature 1B, 7 or 14, which are strongly related to melanoma, while those of ATM-deficient iPSCs resemble to Signature 5 and 9, lymphoma-related signatures (Fig. 5). It is noteworthy that the top five mutation types were all C-to-T transition at dipyrimidine site. Most of other frequent ones also occurred in a dipyrimidine context.

### Increased sensitivity to UV exposure in XPA-iPSCs

**Figure 6:**
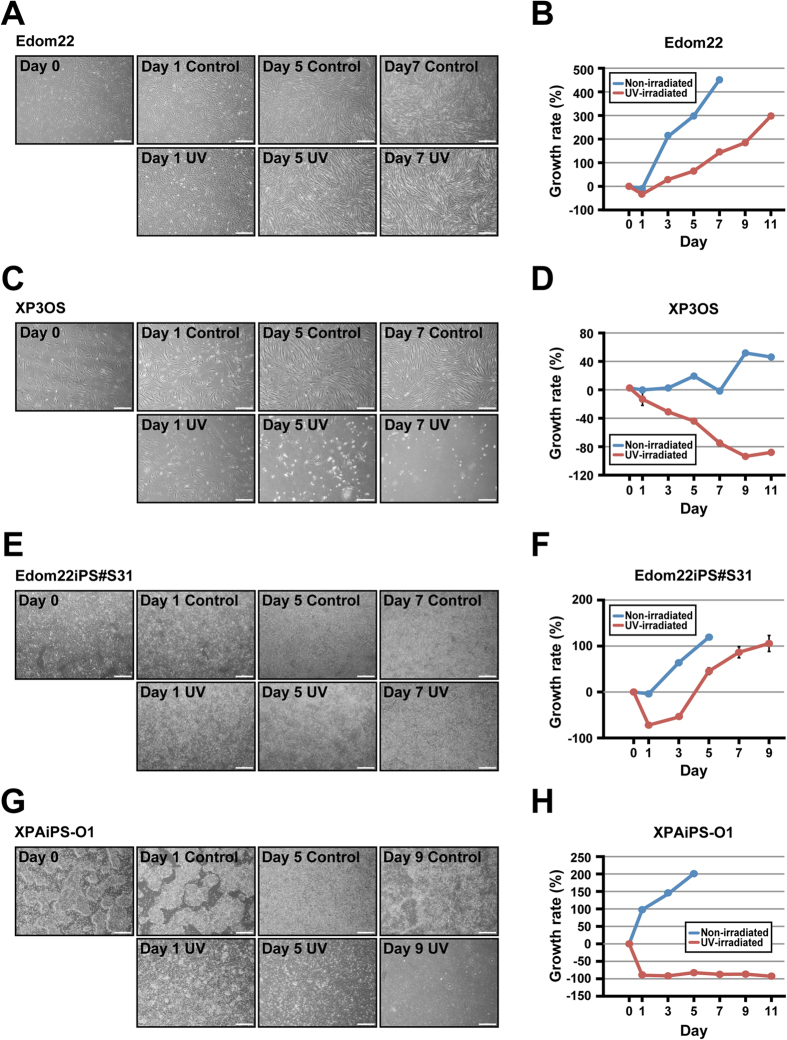
Effect of UV irradiation on Edom22, XP3OS, and their iPSCs. To investigate the effect of UV irradiation on cell morphology, cells were plated into Matrigel-coated 6-well dishes. The cells were exposed to UV irradiation (312 nm at 14 mj/cm^2^) on Day 0. Cell morphology was observed for 7 days. Cell growth was calculated at the indicated days after exposure to UV. Non-irradiated cells were also shown as controls. (**A**) Phase contrast photographs of Edom22 cells on the indicated days after UV irradiation. (**B**) Effect of UV irradiation on growth of Edom22 cells. (**C**) Phase contrast photographs of XP3OS cells on the indicated days after UV irradiation. (**D**) Effect of UV irradiation on growth of XP3OS cells. (**E**) Phase contrast photographs of Edom22iPS#S31 cells on the indicated days after UV irradiation. (**F**) Effect of UV irradiation on growth of Edom22iPS#S31 cells. (**G**) Phase contrast photograph of XPAiPS-O1 cells on the indicated days after UV irradiation. (**H**) Effect of UV irradiation on growth of XPAiPS-O1 cells.

We also investigated the UV sensitivity of XPAiPS-O1 cells and their parental cells. As a reference, we prepared iPSCs from intact Edom22 cells by infecting the same Sendai virus under the same experimental conditions. iPSCs and their parental cells were exposed to UV (14 mj/cm^2^), and then examined for cell growth after UV exposure (Fig. 6). Patient-derived XP3OS cells showed increased UV sensitivity, as compared with intact Edom22 cells. Likewise, XPAiPS-O1 cells exhibited a drastic decrease in cell number and cessation of cell proliferation while healthy donor-derived iPSCs (Edom22iPS#S31) regained growth, albeit with a slight decrease in cell number. These results show that XPA-iPSCs have higher UV sensitivities than the intact iPSCs in growth characteristics.

## Discussion

Although DNA is far more stable than RNA, DNA molecules are also synthesized and decomposed repeatedly in cells. This reminds us that DNA is not chemically inert and that the DNA repair system is indispensable for maintaining life^18^. The system also harnesses the chemically active nature of DNA molecules to repair double-strand breaks or single-strand damage. Combined with the previous study, we have established and characterized several iPSCs related to both damage types not only toward understanding the molecular mechanisms but also toward treatment for DNA repair-deficiency disorders, *i.e.* DNA double-strand break repair deficiency and nucleotide excision repair deficiency.

Ataxia telangiectasia is a hereditary disease characterized by cerebellar ataxia, telangiectasias of the skin, and various severe symptoms in many parts of the body. These patients have an increased risk of cancer due to chromosomal instability, which is caused by a defect in the *ATM* gene^19^. The ATM protein is responsible for recognizing double-strand breaks in DNA. Hence, it is plausible that many structural mutations or chromosomal alterations were identified in the AT1OS-derived iPSCs. XP patients are also known to have a high risk of developing cancer. However, none of structural mutations were detected in the *XPA*-deficient iPSCs in the same method (Table 1). Since there are some reports on occurrence of copy-number mutations during reprogramming^20^, whole-genome resequencing or other high-resolution analyses may be required to detect smaller-scale mutations. Regardless, our contrasting observations obviously reflect the function of the *ATM* gene. It is intriguing that significantly more short indel mutations were found in *ATM*-deficient iPSCs than in *XPA*-deficient iPSCs. This gene might have another unknown function, such as recognition of double-strand distortions caused by an insertion or deletion mutation, in addition to recognizing breaks.

The most surprising result in this study was the vastly increased numbers of single-nucleotide substitutions found in *XPA*-deficient iPSCs. In contrast to *ATM*, the *XPA* gene is responsible for DNA repair by nucleotide excision that targets only a sole nucleotide in one of the two strands^21^. A failure to repair such a lesion results in a single-nucleotide mutation. Nearly a 5-fold increase in point mutations could be attributed to impairment of excision repair. Moreover, up to 43% of all single-nucleotide mutations in the *XPA*-deficient cells were C-to-T or G-to-A transitions, which cannot be distinguished in genomes once replicated. Such a skew was not observed in the *ATM*-deficient cells (Fig. 5). Furthermore, most of the transitions arose at dipyrimidine sites. These features are a typical signature of UV-specific mutations, presumably due to sunlight-induced formation of pyrimidine dimers^22^. Because the *ATM*-deficient iPSCs did not show a high mutation number of C-to-T or G-to-A transitions, most of such transitions identified in the *XPA*-deficient iPSCs might have occurred in the patient before fibroblast sampling. This inference is consistent with recent observations that most of variants identified between an iPSC line and its founder cell line had already existed prior to reprogramming^23^. iPSC clones are thought to be derived from rare founder cells which contained a set of variants compared to the starting bulk. Nevertheless, it is unlikely that only the UV-specific mutations accounting for nearly half of all mutations can give an explanation of the nearly five-fold increase in single-nucleotide mutation numbers in the *XPA*-deficient cells.

Compared to usual whole-exome analyses, our method targeted in total 121 Mb of the genome, letting us detect mutations incurred in non-coding regions (Fig. 4). If we consider numbers of single-nucleotide mutations only in protein-coding regions in the *ATM*-deficient cells, they were comparable to those reported by other groups^23^,^24^,^25^,^26^. Such low level of genetic alterations was also shown in various somatic cell types other than fibroblasts^27^. Because the numbers of single-nucleotide mutations in the *ATM*-deficient cells did not depend on passage number after establishment of the iPSCs (Table 1), the mutations that were detected may be preexisting or have arisen during reprogramming. While many studies concluded a relative stability of iPSCs in spite of the use of oncogenic induction, the numbers of single-nucleotide mutations in the *XPA*-deficient cells were noteworthy (Fig. 2). Unlike in *ATM*-deficient cells, the mutations could also depend on passage number after establishment of the iPSCs, suggesting their higher mutability. To know what proportion of the mutations were preexisting in the precursor cells or arose during reprogramming, additional experiments using different sources may be required. Preferential selection of mutations incurred in non-coding sequences and at synonymous sites was not observed (Fig. 4). Neither of the *XPA*-deficient iPSCs shared any specific mutations.

Although much deeper sequencing or whole-genome sequencing is preferable, the iPSC lines we established can serve as a useful tool for estimation of mutation rates. Due to heterogeneous cell population with different mutations during cultivation, usual cultured cell lines are unsuitable for that purpose. Among a large number of iPSC lines whose mutations were examined, our disease-specific iPSC line sets were shown to retain unique features in terms of mutation type and quantity. They can be used for clarifying more details of DNA repair deficiencies as well as their drug development.

## Materials and Methods

### Ethical statement

Human cells in this study were performed in full compliance with the Ethical Guidelines for Clinical Studies (2008 Notification number 415 of the Ministry of Health, Labor, and Welfare). The cells were banked after approval of the Institutional Review Board at the National Institute of Biomedical Innovation (May 9, 2006). The experimental procedure was approved by the Institutional Review Board (IRB) at National Center for Child Health and Development (IRB number: 827).

### Cells

XP3OS cells were obtained from a 5-year-old Japanese girl (the JCRB Cell Bank, Osaka, Japan). The patient’s history is contained in the original report^28^. The patient developed skin cancer as a 5-year-old and was diagnosed with mental retardation as a 7-year-old. She had a gait disturbance and received Achilles tendon surgery. Her parents have a consanguineous relationship. XPEMB-1 cells were also obtained from the JCRB Cell Bank, Osaka, Japan^29^. The patient history could not be obtained.

XP3OS and XPEMB-1 cells were maintained in the Dulbecco’s modified Eagle’s medium (DMEM, SIGMA D6429) supplemented with 10% or 20% FBS, respectively at 37 °C in a humidified atmosphere containing 95% air and 5% CO_2_. When the cultures reached subconfluence, the cells were harvested with Trypsin-EDTA Solution (cat# 23315, IBL CO., Ltd, Gunma, Japan), and re-plated at a density of 5 × 10^5^ cells in a 100-mm dish. Medium changes were carried out twice a week thereafter.

### Generation of XPA-iPSCs

iPSCs were generated from XP3OS and XPEMB-1 cells through reprogramming by Sendai virus infection-mediated expression of OCT4/3, SOX2, KLF4, and c-MYC as previously described^10^. In addition, iPSCs were also established from menstrual blood-derived cells^30^,^31^ by the same Sendai virus, and Edom22iPS#S31, one of the iPSC clones, was used for comparison. Human iPSCs were maintained on irradiated MEFs as previously described^12^,^32^. Elimination of Sendai virus was confirmed by RT-PCR. Cells just after infection served a positive control. Sequences of the primers set are: forward primer, 5′-AGA CCC TAA GAG GAC GAA GA-3′; reverse primer, 5′-ACT CCC ATG GCG TAA CTC CAT AGT G-3′.

### Immunocytochemical analysis

Cells were fixed with 4% paraformaldehyde in PBS for 10 min at 4 °C. After washing with PBS and treatment with 0.2% Tween 20 in PBS (PBST) for 10 min at 4 °C, cells were pre-incubated with blocking buffer (10% goat serum in PBS) for 30 min at room temperature, and then exposed to primary antibodies in blocking buffer for 12 h at 4 °C (Supplemental Figure S3B). Following washing with 0.2% PBST, cells were incubated with secondary antibodies; either anti-rabbit or anti-mouse IgG conjugated with Alexa 488 or 546 (1:300) (Invitrogen) in blocking buffer for 1 h at room temperature. Then, the cells were counterstained with DAPI and mounted.

### Karyotypic analysis

Karyotypic analysis was contracted out to Nihon Gene Research Laboratories Inc. (Sendai, Japan). Metaphase spreads were prepared from cells treated with 100 ng/mL of Colcemid (Karyo Max, Gibco Co. BRL) for 6 h. The cells were fixed with methanol:glacial acetic acid (2:5) three times, and placed onto glass slides (Nihon Gene Research Laboratories Inc.). Chromosome spreads were Giemsa banded and photographed. A minimum of 10 metaphase spreads were analyzed for each sample, and karyotyped using a chromosome imaging analyzer system (Applied Spectral Imaging, Carlsbad, CA).

### Teratoma formation

XPA-iPSCs were harvested by accutase treatment, collected into tubes, and centrifuged. The cell pellets were suspended in the iPSellon medium. The same volume of Basement Membrane Matrix (354234, BD Biosciences) was added to the cell suspension. The cells (>1 × 10^7^) were subcutaneously inoculated into immuno-deficient, non-obese diabetic (NOD)/severe combined immunodeficiency (SCID) mice (CREA, Tokyo, Japan). The resulting tumors were dissected and fixed with PBS containing 4% paraformaldehyde. Paraffin-embedded tissue was sliced and stained with hematoxylin and eosin (HE). The operation protocols were approved by the Laboratory Animal Care and the Use Committee of the National Research Institute for Child and Health Development, Tokyo.

### Whole-exome sequencing

At passage 10 to 27, genomic DNA was extracted from each cell sample using standard protocols. Each passage number is tabulated in Table 1. Approximately 2.0 μg of each DNA sample was sonicated on a Covaris S220 instrument to produce random short fragments. After several cycles of PCR amplification, capture for target enrichment and library preparation were carried out with Agilent SureSelect Human All Exon V4 + UTRs + lincRNA (80 Mb), followed by washing, elution, and additional PCR amplification. Enriched fragment libraries were then sequenced on an Illumina HiSeq 1000 in 101-bp paired-end mode. Image analyses and base calling were performed using CASAVA 1.8.2 with default parameters.

### Short read mapping and variant detection

Short reads obtained from the sequencer were processed, mapped, and analyzed as previously described by our research group^9^. In brief, the paired-end reads were first trimmed by removing library adapters and low quality bases at ends and then aligned to the hs37d5 sequence (GRCh37 and decoy sequences). Uniquely mapped read pairs were selected to make SAM and BAM files, followed by removal of PCR duplicates, local realignment, recalibration of map quality scores. Multi-sample calling with a precursor cell sample and its iPSC sample was employed for detection of mutations occurred in the iPSCs. Our stringent filter setting was described in detail in the previous report. Annotations of altered sites were made using the ANNOVAR software based on GRCh37^33^.

### Structural alteration analysis using an SNP-genotyping microarray

The structural alteration analysis by genome-wide SNP genotyping was performed using Illumina HumanCytoSNP-12 v2.1 DNA Analysis BeadChip Kit. The microarray contains approximately 300,000 SNP markers (roughly one every 10 kb) with an average call frequency of more than 99%. For each sample, 200 ng of DNA was used as an input for a single array. Whole-genome amplification, fragmentation, hybridization, and allele detection were performed according the manufacturer’s protocol. Subsequent computational and manual analyses were carried out using the Illumina KaryoStudio software.

### UV irradiation

iPSCs were plated into Matrigel-coated 6-well dishes without feeder cells at a density of 3 × 10^5^ cells. When the cells reached a density of 1 × 10^6^, medium was removed, PBS (2 ml) was added, and the dish cover was removed. The cells were then exposed to UV (312 nm at 14 mj/cm^2^) using the WUV-M20 UV transilluminator (AE-6932, ATTO Co.).

## Additional Information

**Accession codes:** The read data have been submitted to the Sequence Read Archive (SRA) under accession number SRP058607. The microarray data have been submitted to the GEO repository under accession number GSE55520.

**How to cite this article**: Okamura, K. *et al*. Distinctive features of single nucleotide alterations in induced pluripotent stem cells with different types of DNA repair deficiency disorders. *Sci. Rep.* **6**, 26342; doi: 10.1038/srep26342 (2016).

## Supplementary Material

Supplementary Information

Supplementary Table S1

Supplementary Table S2

## Figures and Tables

**Table 1:**
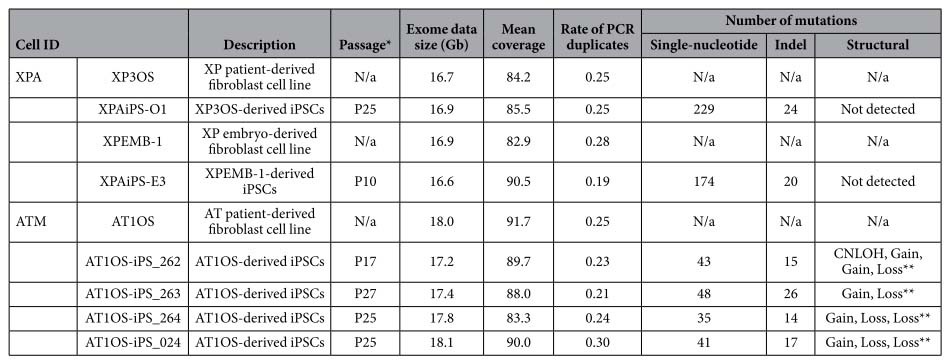
List of human cells analyzed for mutations.

*Number of passages after establishment of iPSCs. **The locations are tabulated in a previous study (Fukawatase *et al*.^9^). “N/a” stands for not applicable.
