# Supplementary material for: Distinctive features of single nucleotide alterations in induced pluripotent stem cells with different types of DNA repair deficiency disorders
Source: Sci Rep. 2016 May 20;6:26342. doi: 10.1038/srep26342 (PMC4873825; doi:10.1038/srep26342)
Supplement: Supplementary Information [file srep26342-s1.pdf]

Supplementary Information

Distinctive features of single nucleotide alterations in induced pluripotent stem cells with different types of DNA repair deficiency disorders

Kohji Okamura, Hironari Sakaguchi, Rie Sakamoto-Abutani, Mahito Nakanishi, Ken Nishimura, Mayu Yamazaki-Inoue, Manami Ohtaka, Vaiyapuri Subbarayan Periasamy, Ali Abdullah Alshatwi, Akon Higuchi, Kazunori Hanaoka, Kazuhiko Nakabayashi, Shuji Takada, Kenichiro Hata, Masashi Toyoda, and Akihiro Umezawa

A

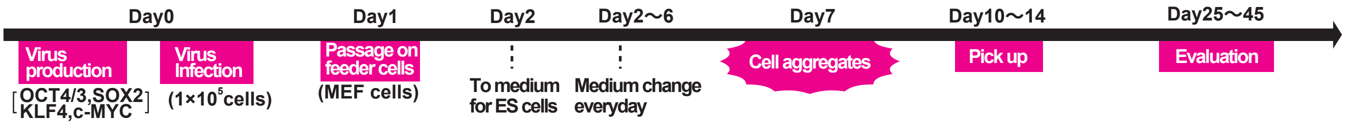

B

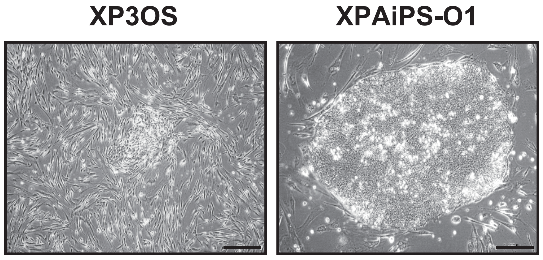

C

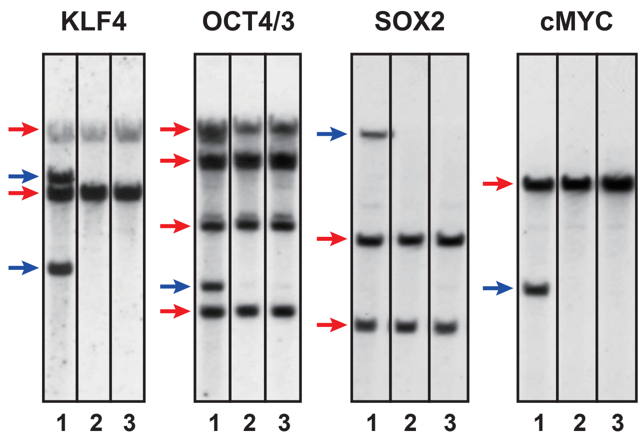

Copy number of the transgenes

Lane No.	Sample	KLF4	OCT4/3	SOX2	cMYC
1	MRCiPS-25	2	1-2	1	1
2	XP3OS	0	0	0	0
3	XPAiPS-O1	0	0	0	0

D

Locus	XP3OS		XPAiPS-O1	
D3S1358	15	16	15	16
TH01	7		7	
D21S11	29	30	29	30
D18S51	13		13	
Penta_E	14	17	14	17
D5S818	10	11	10	11
D13S317	9	11	9	11
D7S820	11	12	11	12
D16S539	9	12	9	12
CSF1PO	10	11	10	11
Penta_D	9		9	
AMEL	X		X	
vWA	14	16	14	16
D8S1179	13	15	13	15
TPOX	8	11	8	11
FGA	22	23	22	23

Supplemental Figure S1. Generation of iPS cells from xeroderma pigmentosum-derived cells
(A) Protocol for iPSC generation. **(B)** Phase-contrast image of XP3OS and XPAiPS-O1 cells. **(C)** Southern blot analysis using the probes for the transgenes. Transgenes were identified by Southern blotting analysis of MRCiPS-25, XP3OS, and XPAiPS-O1 cells with the probes for KLF4 (whole cDNA), OCT4/3 (383 bp from the start site), SOX2 (424 bp from the stop codon), and c-MYC (whole cDNA). Copy numbers were determined by a bio-imaging analyzer, using copy number controls. Blue and red arrows indicate transgenes and endogenous genes, respectively. The extra bands of the OCT4/3 endogenous gene are likely derived from the endogenous pseudogenes (Jez M. et al, PLoS One. 9:e89546, 2014). **(D)** STR analysis of XP3OS and XPAiPS-O1 cells. Short tandem repeat (STR) profiling was performed by BEX CO., LTD, Tokyo, Japan. The 16 loci analyzed by the PowerPlex 1.2 system (Promega, Madison, WI, USA) was composed of D3S1358, TH01, D21S11, D18S51, Penta E, D5S818, D13S317, D7S820, D16S539, CSF1PO, Penta D, AMEL, vWA, D8S1179, TPOX, and FGA.

Supplemental Figure S1

XP3OS

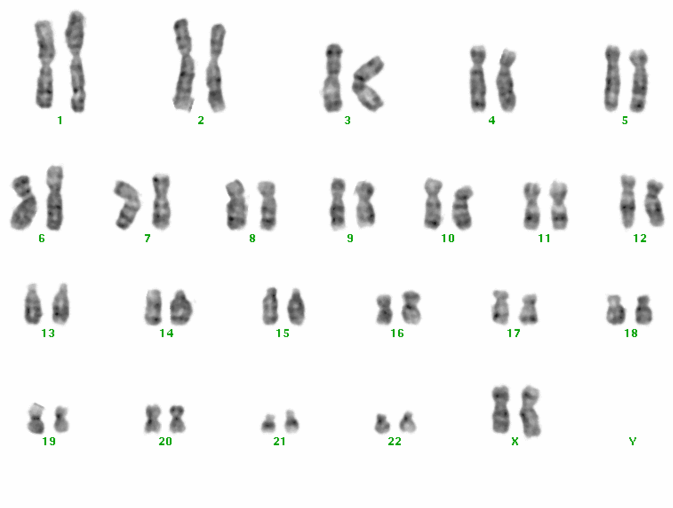

46, XX [20]

XPAiPS-O1

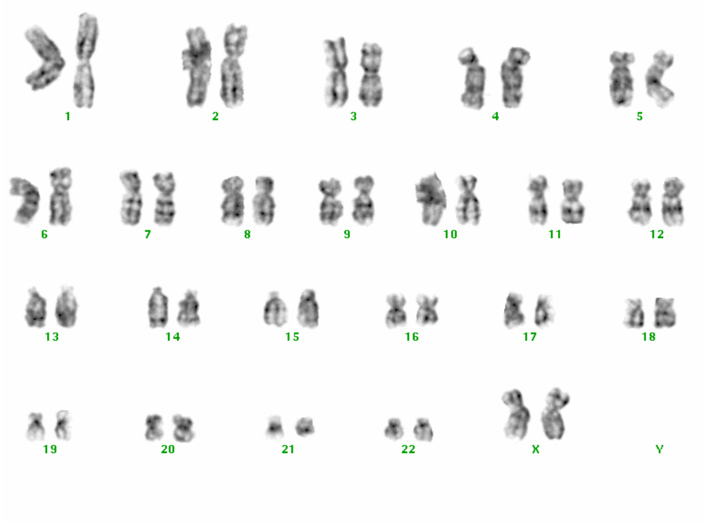

46, XX [20]

Supplemental Figure S2. Karyotypic analysis of the parental XP3OS cells and XPAiPS-O1 cells
In total, 20 cells were analyzed and the number of cells with the indicated karyotype was shown in brackets.

A

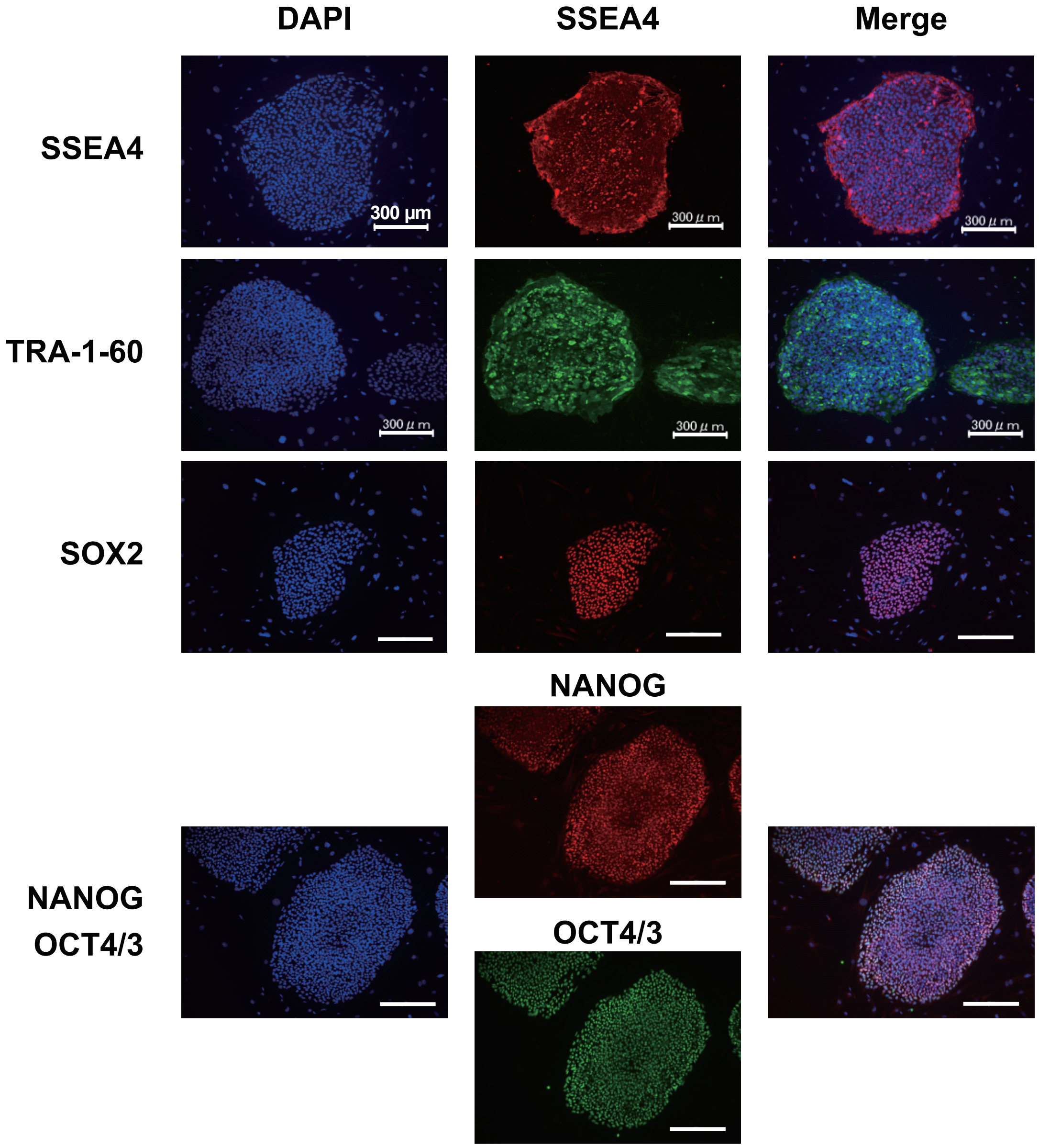

B

Antibodies for immunocytochemistry			
1st antibody	Product name	Cat.no., maker	Dilution
SSEA4	Ms X SSEA-4, IgG, clone MC-813-70	MAB4304, Millipore	1/300
TRA-1-60	Ms X TRA-1-60, IgM, clone TRA-1-60	MAB4360, Millipore	1/300
OCT4/3	Oct3/4(c-10), mouse monoclonal IgG2b	sc-5279, Santa Cruz	1/300
NANOG	Anti Human Nanog Polyclonal antibody	RCAB0003P, Repro Cell	1/300
SOX2	Rabbit anti-Sox2 affinity purified polyclonal antibody	AB5603, Millipore	1/300
2nd antibody	Product name	Cat.no., maker	Dilution
SSEA4	Alexa Fluor 488 F(ab')2 fragment of goat anti-mouse IgG(H+L)	A11017, Invitrogen	1/300
TRA-1-60	Alexa Fluor 546 goat anti-mouse IgM (m chain)	A21045, Invitrogen	1/300
OCT4/3	Alexa Fluor 546 goat anti-mouse IgG2b (g2b)	A21143, Invitrogen	1/300
NANOG	Alexa Fluor 488 goat anti-rabbit IgG(H+L)	A11008, Invitrogen	1/300
SOX2	Alexa Fluor 488 goat anti-rabbit IgG(H+L)	A11008, Invitrogen	1/300
DAPI	DAPI, 40043, Biotium	40043, Biotium	1/300

Supplemental Figure S3. Immunocytochemical analysis of XPAiPS-O1 cells
(A) Immunocytochemical analysis using antibodies to SSEA-4, TRA-1-60, SOX2, NANOG, and OCT4/3 used. **(B)** Antibodies used for immunocytochemistry.

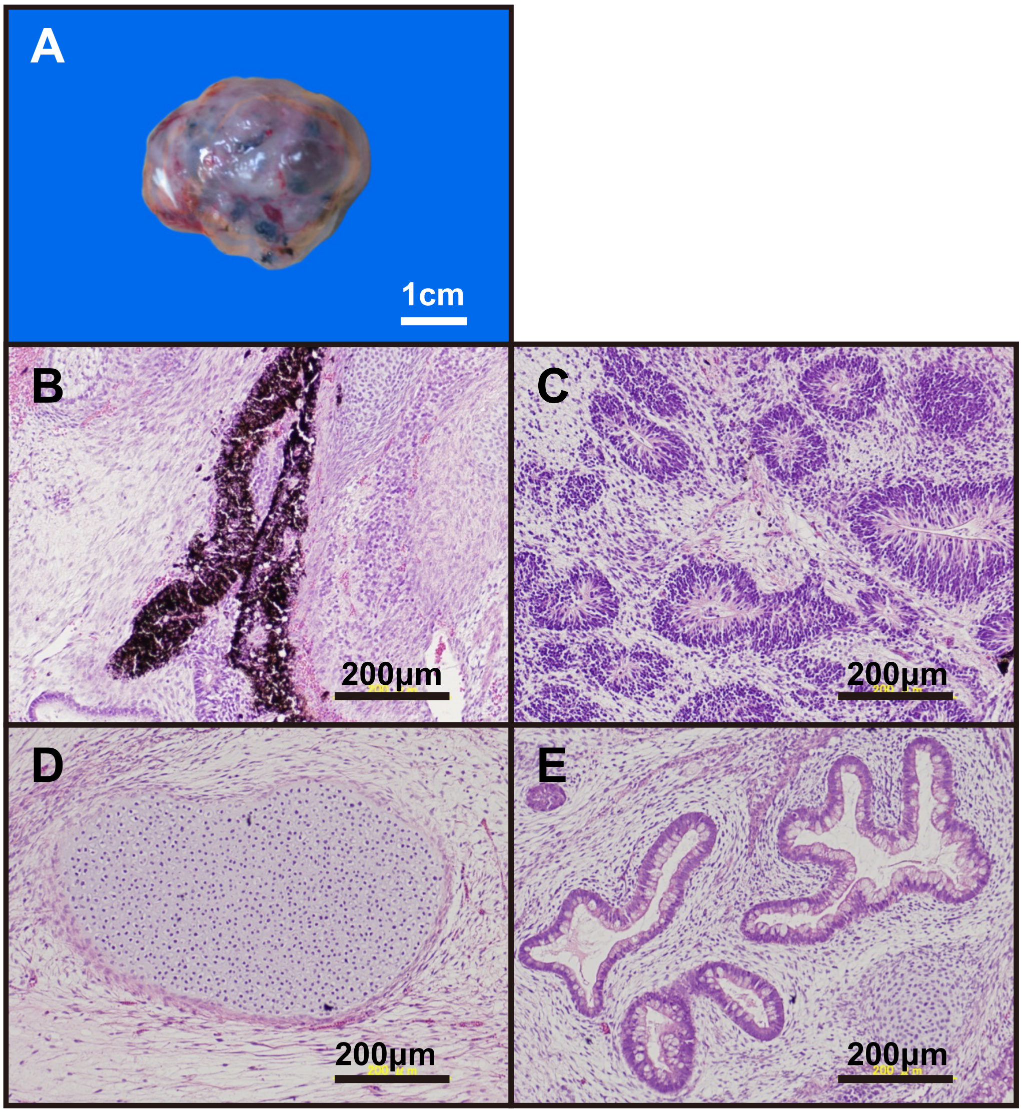

Supplemental Figure S4. Histological analysis of teratomas generated by XPA-iPSC clones
Teratoma formation was observed within 6-10 weeks after injection of XPAiPS-O1 cells into immunodeficient mice (**A**: macroscopic view of a teratoma extracted from a mouse, **B**: retinal pigment epithelium, **C**: neuroectodermal tissue, **D**: cartilage, **E**: intestine).

A

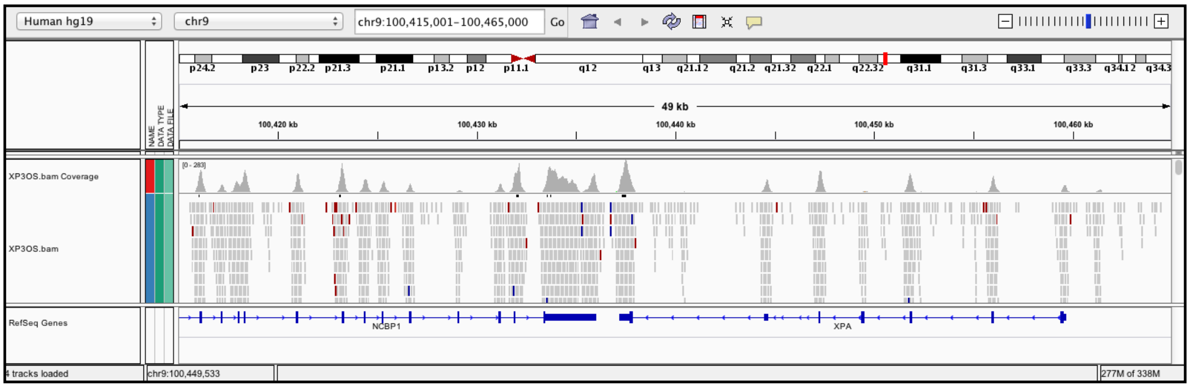

B

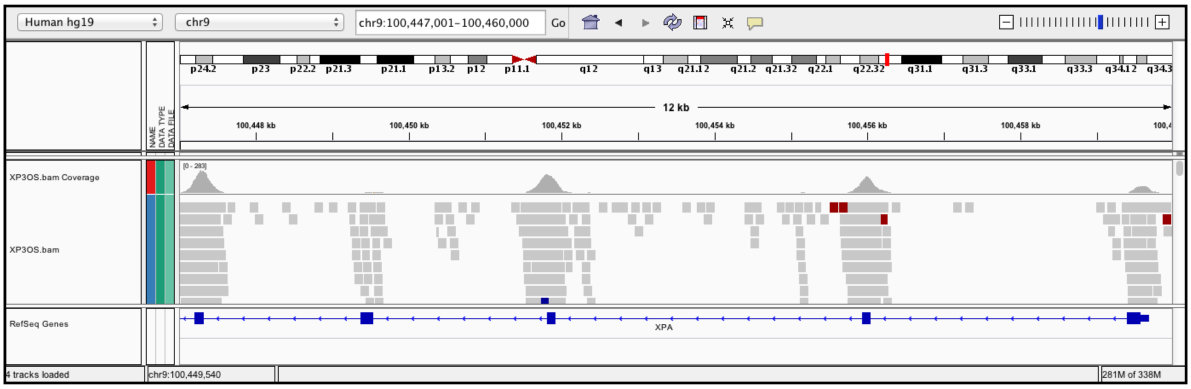

C

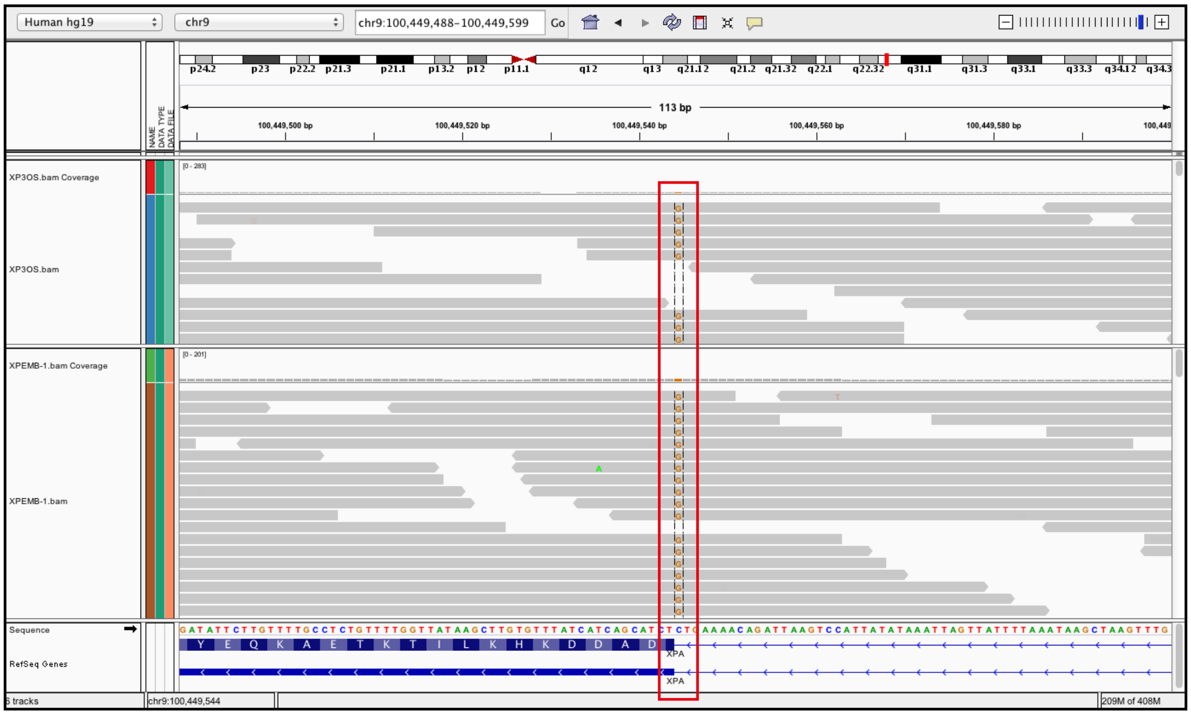

Supplemental Figure S5. XPA locus and the homozygous mutation at a splice accepter site (A) The XPA gene is encoded on the complementary strand of 9q22.3. **(B)** Short reads mapped to the exons 1 to 5 of the XPA gene are shown as gray bars. The homozygous mutation (arrowheads) was found in both the XP3OS and XPEMB-1 cells. **(C)** The homozygous mutation (arrowheads) was found in both the XP3OS and XPEMB-1 cells. These snapshots were obtained by using Integrative Genomics Viewer (https://www.broadinstitute.org/igv/) (Thorvaldsdóttir et al., 2013).

Supplemental Figure S5
